# Bone marrow-derived mesenchymal stem cells ameliorate chronic high glucose-induced *β*-cell injury through modulation of autophagy

**DOI:** 10.1038/cddis.2015.230

**Published:** 2015-09-17

**Authors:** K Zhao, H Hao, J Liu, C Tong, Y Cheng, Z Xie, L Zang, Y Mu, W Han

**Affiliations:** 1 Department of Endocrinology, Chinese PLA General Hospital, Beijing, China; 2 Department of Molecular Biology, Institute of Basic Medicine, College of Life Science, Chinese PLA General Hospital, Beijing, China

## Abstract

Chronic hyperglycemia causes a progressive decrease of *β*-cell function and mass in type 2 diabetic patients. Growing evidence suggests that augment of autophagy may be an effective approach to protect *β* cells against various extra-/intracellular stimuli. In this study, we thus investigated whether bone marrow-derived mesenchymal stem cells (BM-MSCs) could ameliorate chronic high glucose (HG)-induced *β*-cell injury through modulation of autophagy. Prolonged exposure to HG decreased cell viability, increased cell apoptosis and impaired basal insulin secretion and glucose-stimulated insulin secretion of INS-1 cells, but BM-MSC treatment significantly alleviated these glucotoxic alternations. In addition, western blotting displayed upregulated expression of Beclin1 and LC3-II in INS-1 cells co-cultured with BM-MSCs. Results from immunofluorescence staining and transmission electronic microscope analysis also revealed that BM-MSCs promoted autophagosomes and autolysosomes formation in HG-treated INS-1 cells. However, it should be noted that inhibition of autophagy significantly diminished the protective effects of BM-MSCs on HG-treated INS-1 cells, suggesting that the improvement of *β*-cell function and survival induced by BM-MSCs was mediated through autophagy. Furthermore, our results showed that BM-MSCs improved mitochondrial function and reduced reactive oxygen species production in HG-treated INS-1 cells, largely owing to autophagic clearance of impaired mitochondria. *In vivo* study was performed in rats with type 2 diabetes (T2D). BM-MSC infusion not only ameliorated hyperglycemia, but also promoted restoration of pancreatic *β* cells in T2D rats. Meanwhile, BM-MSC infusion upregulated LAMP2 expression and enhanced formation of autophagosomes and autolysosomes, combined with reduced *β*-cell apoptosis and increased number of insulin granules. These findings together indicated that BM-MSCs could protect *β* cells against chronic HG-induced injury through modulation of autophagy *in vitro* and *in vivo*. This study unveiled novel evidence of BM-MSCs as an ideal strategy to enhance autophagy for treatment of T2D mellitus.

Type 2 diabetes (T2D) mellitus, the most common metabolic disorder in humans, is characterized by hyperglycemia resulting from pancreatic *β*-cell failure and inadequate insulin secretion to compensate for insulin resistance. In spite of intensive therapy, it is inevitable to witness *β*-cell deterioration in type 2 diabetic patients with the advancing duration of disease because oral hypoglycemic drugs and exogenous insulin are sometimes insufficient to normalize blood glucose levels or achieve sustained glycemic control.^1, 2, 3^ The resultant chronic hyperglycemia is a major cause of progressive decline in *β*-cell function and mass.^1, 3, 4^ Ample evidence has demonstrated that prolonged exposure to elevated glucose concentrations induces a series of glucotoxic alterations in *β* cells, such as oxidative stress, endoplasmic reticulum (ER) stress and protein glycation, leading to irreversible *β*-cell dysfunction and eventual apoptosis.^3, 4, 5^ Therefore, novel avenues that can not only improve glycemic control but also prevent *β*-cell deterioration during chronic hyperglycemia would be valuable therapeutic strategies to treat T2D.

Mesenchymal stem cells (MSCs), one class of pluripotent cells that are capable of differentiating into multi-lineage cells,^6^ counteracting autoimmunity^7^ and secreting various cytokines and growth factors,^8^ have exhibited significant anti-diabetic effects in animal studies and clinical trials.^9, 10, 11^ Research conducted by Liu *et al.*^12^ indicated that, treatment with Wharton's Jelly-derived MSCs improved metabolic control and *β*-cell function in patients with T2D mellitus. Furthermore, studies in diabetic animals revealed that MSC treatment reduced blood glucose levels and regenerated pancreatic islets and *β* cells to varying degrees.^13, 14^ Nevertheless, the precise mechanisms underlying these effects could not be adequately explained by MSC transdifferentiation or immunomodulation. In our previous studies, we demonstrated that bone marrow (BM)-MSC infusion significantly ameliorated hyperglycemia through improved insulin sensitivity; and the results also showed that BM-MSCs promoted restoration of pancreatic islets and *β* cells in T2D rats, whereas the ‘increased' islet and *β*-cell numbers were not generated by cell proliferation. We therefore proposed that the impact on pancreatic islets and *β*-cell recovery may be largely caused by the cytoprotective properties of infused MSCs.^15^ However, further studies are required to test this hypothesis.

Macroautophagy, referred to here as autophagy, is a dynamic process in which double-membrane autophagosomes engulf cellular components (e.g., damaged organelles and toxic proteins) and fuse with lysosomes for degradation and recycling of their nutrients and constituents.^16, 17^ Autophagy has a well-established and indispensable role in a variety of diseases, including neurodegenerative disorders, cardiovascular diseases, cancer and infectious diseases.^18, 19^ The importance of autophagy has recently been appreciated in the pathogenesis of T2D.^20, 21^ It has been reported that in pancreatic *β* cells of type 2 diabetic patients, altered autophagy occurred with hampered removal of autophagic material, reduced expression of lysosome-associated membrane protein 2 (LAMP2) and of cathepsin B and D.^22^ In animal studies, several lines of evidence has suggested that basal autophagy is essential to maintain the architecture and function of pancreatic *β* cells, whereas deficient autophagy impairs *β*-cell mass and function to further exacerbate the pathogenesis of T2D.^23, 24^ Conversely, enhancement of autophagy exerted cytoprotective effects on *β* cells under adverse conditions.^25, 26^ Bachar-Wikstrom *et al.*^25^ demonstrated that stimulation of autophagy prevented *β*-cell apoptosis and improved ER stress-induced diabetes in Akita mice. Most importantly, recent research indicated that MSCs stimulated autophagy and cleared accumulated toxic proteins, contributing to increased neuronal survival in neurodegenerative disorders.^27, 28^ However, there is currently no evidence describing the potential impacts of MSCs on autophagy in the dysfunctional *β* cells of T2D.

Mitochondria have an imperative role in glucose-stimulated insulin secretion (GSIS) and *β*-cell survival.^29, 30^ During chronic hyperglycemia, impaired mitochondria leads to considerable production of reactive oxygen species (ROS), which in turn aggravates mitochondrial damage and other intracellular abnormalities, contributing to *β*-cell dysfunction and apoptosis.^31^ However, several studies have reported that autophagy can remove damaged organelles, such as mitochondria, as an adaptive response to unfavorable circumstances. Dutta *et al.*^32^ revealed that increased autophagy offered cytoprotection against oxidative stress via clearance of dysfunctional mitochondria in cardiomyocytes. Similar protective effects of autophagy were also verified in endothelial cells^33^ and osteoblasts.^34^ Thus, augment of autophagy for eliminating impaired mitochondria appears to be an effective approach to protect *β* cells against chronic high glucose (HG)-induced injury.

In this study, INS-1 cells were chronically exposed to HG medium, and T2D was induced using a high-fat diet/STZ in rats. Our results showed that BM-MSCs enhanced autophagy and thereby protect *β* cells against chronic HG-induced injury *in vitro*, and *in vivo*, the autophagic activity of pancreatic *β* cells could be modulated by BM-MSC infusion in T2D rats. This study may provide novel and important evidence supporting future clinical use of MSC therapy for T2D.

## Results

### Identification of BM-MSC characteristics

BM-derived cells at passage 3 were used to co-culture with INS-1 cells, we therefore identified whether these cells had the characteristics of MSCs through measurement of their phenotypes and multiple differentiating capacities. As shown in Supplementary Figures 1a–c, BM-derived cells were able to differentiate into adipogenic and osteoblastic lineages under certain appropriate conditions. On the other hand, results from flow cytometric analysis revealed that BM-derived cells were positive for CD29, CD44 and CD105, whereas negative for CD14, CD34 and CD45 (Supplementary Figure 1d). These data indicated that the BM-derived cells that we used in the following experiments possessed the characteristics of MSCs.

### BM-MSCs alleviated chronic HG-induced injury in INS-1 cells

**Figure 1:**
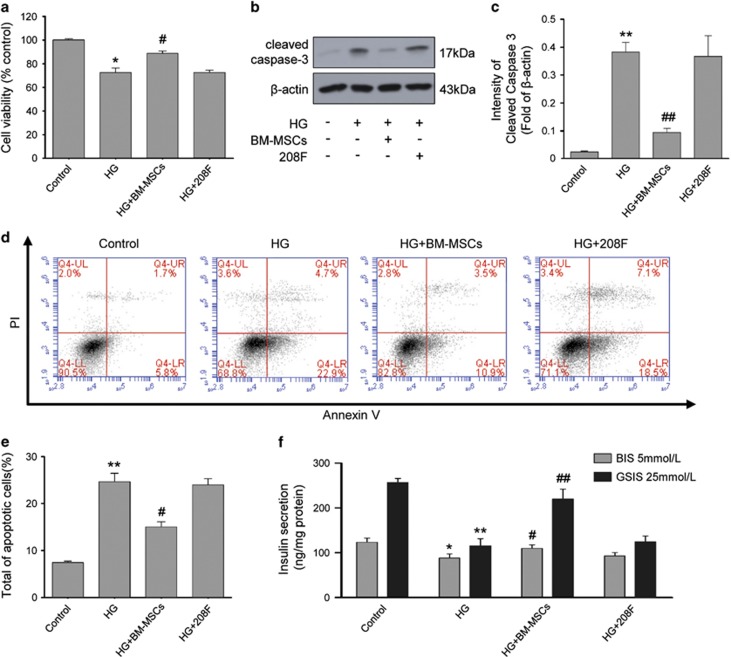
BM-MSCs protected INS-1 cells against chronic HG-induced injury. (**a**) Cellular viability was determined by CCK-8 assay. The data are expressed as percentages of untreated control cells. (**b** and **c**) Western blot analysis of cleaved caspase 3. Protein expression levels were normalized against *β*-actin. (**d** and **e**) Apoptosis incidence in INS-1 cells was detected by Annexin V-FITC/PI double staining. Cells at early stages of apoptosis were Annexin V-FITC positive but PI negative. Cells at late stage of apoptosis were both Annexin V-FITC and PI positive. These two stages combined measurements were counted as total apoptotic cells. (**f**) Insulin secretion levels at basal (5 mmol/l) or stimulatory (25 mmol/l) concentrations of glucose were determined using an insulin RIA kit and normalized to the respective protein contents. Results are means±S.E. of five independent experiments. *P<0.05, **P<0.01 *versus* control group; ^#^*P*<0.05, ^##^*P*<0.01 *versus* HG group

As chronic HG is toxic and deleterious to *β*-cell survival and function,^3, 4^ we investigated whether BM-MSCs could protect INS-1 cells against chronic HG-induced injury. First, we measured cell viability using cell counting kit-8 (CCK-8) assay, and the results showed that chronic exposure of INS-1 cells to HG decreased cell viability compared with untreated control cells, but BM-MSC treatment significantly improved this alternation (Figure 1a). In addition, western blot analysis exhibited upregulated expression of cleaved caspase 3 (a commonly used marker of apoptosis) in chronic HG-treated INS-1 cells, whereas their apoptotic incidence detected by Annexin V/propidium iodide (PI) staining and flow cytometer was also highly increased. However, BM-MSC co-culture significantly downregulated the expression of cleaved caspase 3 and reduced the apoptotic incidence, indicating that BM-MSCs could inhibit INS-1 cell apoptosis induced by chronic HG (Figures 1b–e). We next examined basal insulin secretion (BIS) and GSIS to investigate the effects of BM-MSCs on *β*-cell function. Our results displayed that prolonged culture of INS-1 cells in HG medium impaired BIS and GSIS, but BM-MSC treatment significantly preserved INS-1 cell function, as reflected by a marked increase in BIS and GSIS (Figure 1f). Interestingly, there was no significant change in those parameters mentioned above, when 208F cells, a group of rat fibroblasts, were co-cultured with HG-treated INS-1 cells, suggesting that BM-MSCs had their distinct characteristics and great potential to protect INS-1 cells against chronic HG-induced injury, whereas 208F cells did not have (Figures 1a–f). These findings together revealed that BM-MSCs prevented *β*-cell apoptosis and dysfunction induced by chronic HG, exerting their cytoprotective effects on *β* cells.

### BM-MSCs enhanced autophagy in chronic HG-treated INS-1 cells

**Figure 2:**
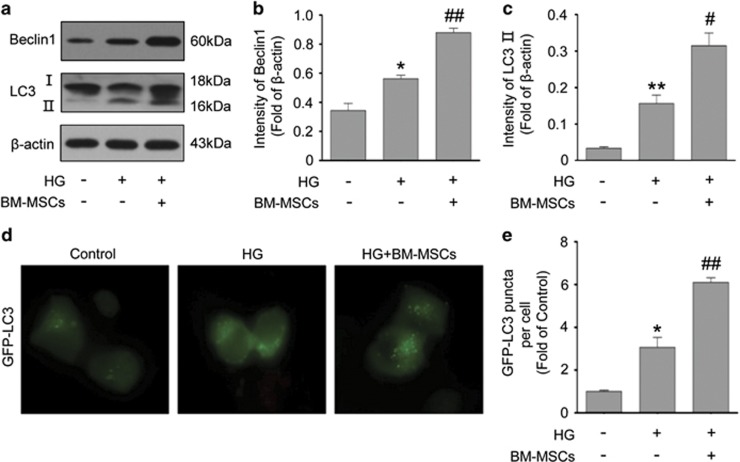
BM-MSCs promoted the formation of autophagosomes. (**a-c**) Western blot evaluation of Beclin1 and LC3-II. Protein expression levels were normalized against *β*-actin. (**d** and **e**) INS-1 cells were transiently transfected with GFP-LC3 plasmid to quantify autophagy. Autophagy was quantified by counting the GFP-LC3 puncta in INS-1 cells. Scale bar, 10 *μ*m. Values are means±S.E. (*n*=5) from independent experiments. *P<0.05, **P<0.01 *versus* control group; ^#^*P*<0.05, ^##^*P*<0.01 *versus* HG group

Growing evidence supports that autophagy has an important protective role in resistance to stress or injury in disease states.^35, 36^ To determine if BM-MSCs impacted autophagy in INS-1 cells under chronic HG conditions, we measured the expression of two autophagic markers, Beclin1 (Atg6) and microtubule-associated protein 1 light chain 3 (LC3, also known as Atg8). Beclin1 is involved in the early phase of autophagosome formation. LC3 is widely used to monitor autophagy; and type II of LC3 (LC3-II), which is converted from type I of LC3 (LC3-I), serves as a typical marker of completed autophagosomes as it is tightly associated with autophagosomes membranes. As shown in Figures 2a–c, there were increased expression of Beclin1 and LC3-II in INS-1 cells chronically exposed to HG. Nonetheless, we surprisingly found that BM-MSC treatment led to much higher levels of Beclin1 and LC3-II in HG-treated INS-1 cells, suggesting the enhanced autophagosomesformation. To confirm our western blotting results, INS-1 cells were transiently transfected with green fluorescent protein (GFP)-LC3 plasmid and the autophagosomes was quantified by counting the GFP-LC3 puncta. We found that HG-treated INS-1 cells displayed an increase in the formation of GFP-LC3 puncta, whereas BM-MSC co-culture caused further increased number of GFP-LC3 punctate staining, also suggesting the enhancement in autophagosomes formation (Figures 2d and e).

**Figure 3:**
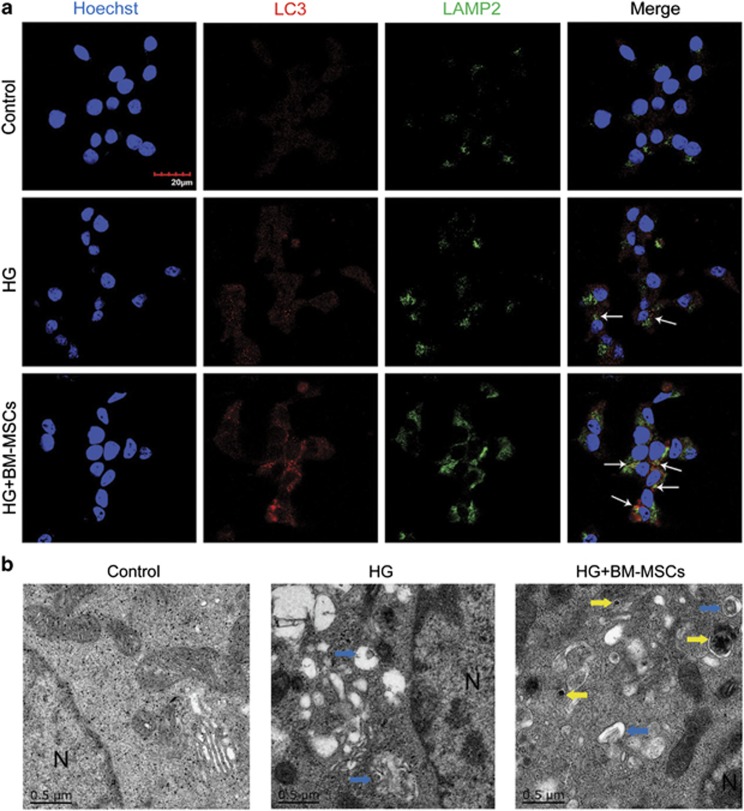
BM-MSCs significantly enhanced the formation of autophagosomes and autolysosomes. (**a**) Immunofluorescence analysis was used to assess the number of LC3-positive (red) autophagosomes colocalized with LAMP2-labeled (green) lysosomes. White arrows point at the colocalization. Scale bar, 20 *μ*m. (**b**) TEM analysis showed ultrastructural changes in INS-1 cells. N, nucleus. Blue arrows, autophagosomes; yellow arrows, autolysosomes. Scale bar, 0.5 *μ*m. Magnification: × 40 000. All the data were collected from at least three independent experiments

However, it is noteworthy that a completed process of autophagy requires the formation of autolysosomes for degradation through fusion of autophagosomes and lysosomes. To determine whether BM-MSCs also enhanced autolysosomes formation, we performed immunofluorescence analysis and transmission electron microscope (TEM) analysis. Initially, immunofluorescence analysis displayed that BM-MSCs raised the number of LC3-positive autophagosomes being colocalized with LAMP2-labeled lysosomes, compared with little presence of fusion between autophagosomes and lysosomes in INS-1 cells treated with HG alone, indicating increased autolysosomes formation caused by BM-MSCs (Figure 3a). In addition, TEM analysis revealed that there were few autophagosomes and rare autolysomes in INS-1 cells cultured in HG medium, but BM-MSC co-culture greatly increased the number of autophagosomes and autolysosomes, which is consistent with our immunofluorescence results (Figure 3b). Furthermore, we discovered that the autophagic activity in HG-treated INS-1 cells could also be promoted by the addition of BM-MSC-conditioned medium (CM), but when INS-1 cells were co-cultured with 208F cells, there was no significant change in autophagic activity (compared with INS-1 cells treated with HG alone) (Supplementary Figure 2).

Overall, these findings demonstrated that although prolonged exposure of INS-1 cells to HG enhanced autophagosomes formation to a certain extent, there was lack of fusion of autophagosomes and lysosomes; however importantly, BM-MSCs significantly promoted the formation of both autophagosomes and autolysosomes in chronic HG-treated INS-1 cells, which maybe largely caused by the secretive properties of BM-MSCs.

### Inhibition of autophagy diminished the protective effects of BM-MSCs on chronic HG-treated INS-1 cells

**Figure 4:**
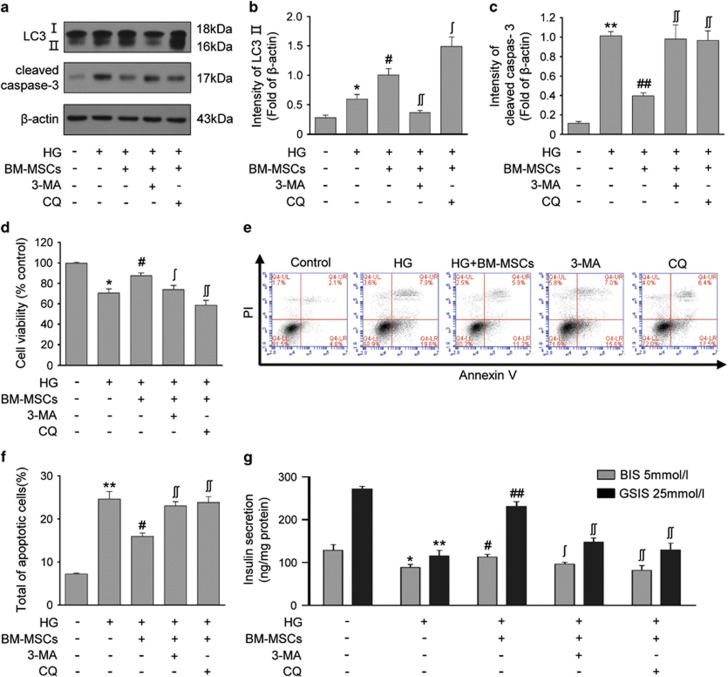
Inhibition of autophagy diminished the protective effects of BM-MSCs on HG-treated INS-1 cells. INS-1 cells co-cultured with BM-MSCs were treated with 3-MA (10 nM) and CQ (20 *μ*M). (**a** and **b**) 3-MA reduced LC3-II expression by inhibiting class III PI3K at an early stage of autophagy; CQ led to additional accumulation of LC3-II by inhibiting lysosome protease at a late stage of autophagy. 3-MA and CQ treatment both resulted in a significant reduction of cell viability (**d**), upregulated expression of cleaved caspase 3 (**a** and **c**), increased incidence of apoptosis (**e** and **f**) and impaired BIS and GSIS (**g**). Results are means±S.E. of five separate experiments. *P<0.05, **P<0.01 *versus* control group; ^#^*P*<0.05, ^##^*P*<0.01 *versus* HG group; ^#^*P*<0.05, ^##^*P*<0.01 *versus* HG+BM-MSCs group

To determine whether the protective effects of BM-MSCs on HG-treated INS-1 cells are mediated through autophagy, 3-methyladenine (3-MA) and chloroquine (CQ), two extensively used autophagy inhibitors, were respectively added in the culture medium for suppressing autophagic process in INS-1 cells. As shown in Figures 4a and b, 3-MA, which inhibits class III phosphatidylinositol 3-kinase to block autophagosomes formation at the early stage of autophagy, significantly downregulated the expression of LC3-II in BM-MSC-co-cultured INS-1 cells. Although CQ, a lysosome protease inhibitor that blocks the late stage of autophagy, induced an additional LC3-II accumulation. These data showed the efficiency of 3-MA and CQ for inhibiting autophagy and further confirmed that BM-MSCs promoted autophagic activity in chronic HG-treated INS-1 cells. In addition, we discovered that inhibition of autophagy with 3-MA or CQ significantly reduced cell viability, upregulated the expression of cleaved caspase 3 and increased the number of apoptotic cells as compared with INS-1 cells co-cultured with BM-MSCs (Figures 4a and c–f). Meanwhile, the addition of 3-MA or CQ also significantly impaired BIS and GSIS in BM-MSCs co-cultured INS-1 cells (Figure 4g). Collectively, autophagy inhibition diminished the protective effects of BM-MSCs on *β*-cell function and survival under chronic HG conditions, indicating that BM-MSCs attenuated chronic HG-induced *β*-cell injury through modulation of autophagy.

### BM-MSCs promoted mitochondrial renovation by autophagic clearance of impaired mitochondria in chronic HG-treated INS-1 cells

**Figure 5:**
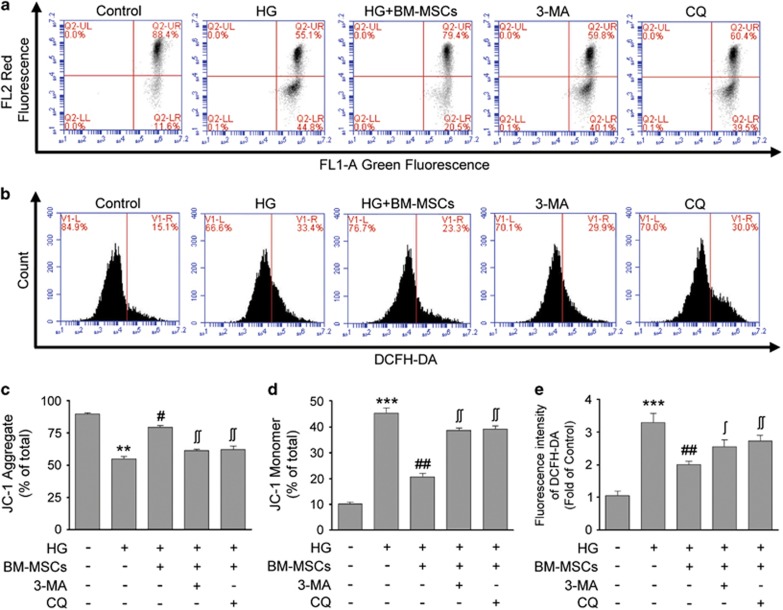
BM-MSCs improved MMP and reduced ROS production through autophagy in chronic HG-treated INS-1 cells. (**a**) MMP was measured using the JC-1 probe by flow cytometry. (**c** and **d**) JC-1 aggregates (cells emitting red fluorescence in the FL2 channel) suggested normal MMP in INS-1 cells. In contrast, accumulation of JC-1 monomers (cells emitting green JC-1 detected in the FL1 channel) indicated MMP depolarization in INS-1 cells. The percentages of INS-1 cells containing JC-1 aggregates or JC-1 monomers were respectively shown in the histograms. (**b** and **e**) Intracellular ROS level was stained with DCFH-DA and detected by flow cytometry. BM-MSCs reduced ROS production in INS-1 cells cultured in a HG medium, but this effect could be suppressed by 3-MA and CQ. Data are expressed as means±S.E. of three replicates from five separate experiments. *P<0.05, **P<0.01 *versus* control group; ^#^*P*<0.05, ^##^*P*<0.01 *versus* HG group; ^#^*P*<0.05, ^##^*P*<0.01 *versus* HG+BM-MSCs group

Mitochondrion is not only a key determinant in GSIS but also a crucial contributing factor to *β*-cell survival.^29, 30^ It is well established that chronic HG induces *β*-cell mitochondrial dysfunction. We therefore investigated whether the cytoprotective effects of BM-MSCs were related to the improvement of mitochondrial function in HG-treated INS-1 cells. Mitochondrial membrane potential (MMP), which is indispensable to mitochondrial oxidative phosphorylation and ATP formation, was quantified using fluorescent mitochondrial probe JC-1 and flow cytometer. MMP depolarization, as reflected by the reduction of JC-1 aggregates and the accumulation of JC-1 monomers, usually occurs in apoptotic cells. Our results displayed that chronic exposure of INS-1 cells to HG resulted in the conversion of JC-1 aggregates into JC-1 monomers as compared with untreated control cells, suggesting that MMP depolarization was induced by chronic HG. However, this alternation was markedly reversed after BM-MSC treatment, as reflected by increased JC-1 aggregates and reduced JC-1 monomers, indicating that BM-MSCs ameliorated HG-induced mitochondrial dysfunction in INS-1 cells (Figures 5a, c and d). To confirm the improvement of mitochondrial function caused by BM-MSCs, intracellular ROS levels in INS-1 cells was served as another parameter of mitochondrial function. Mitochondria are the major source of ROS production; and dysfunctional mitochondria results in excessive generation of ROS, which in turn aggravates mitochondrial impairment resulting from the vulnerability of mitochondria to ROS. As shown in Figures 5b and e, prolonged exposure to HG led to greatly increased ROS levels in INS-1 cells as compared with those in untreated control cells, whereas BM-MSC co-culture significantly reduced the abnormal ROS production in HG-treated INS-1 cells, suggesting the positive effects of BM-MSCs on amelioration of mitochondrial dysfunction. Nonetheless, our results also revealed that the addition of autophagy inhibitors (3-MA or CQ) partially abrogated the beneficial effects of BM-MSCs on MMP and ROS levels in HG-treated INS-1 cells. Thus, we proposed that these improvements of MMP and intracellular ROS levels provided by BM-MSCs were at least partially dependent on autophagic activity.

**Figure 6:**
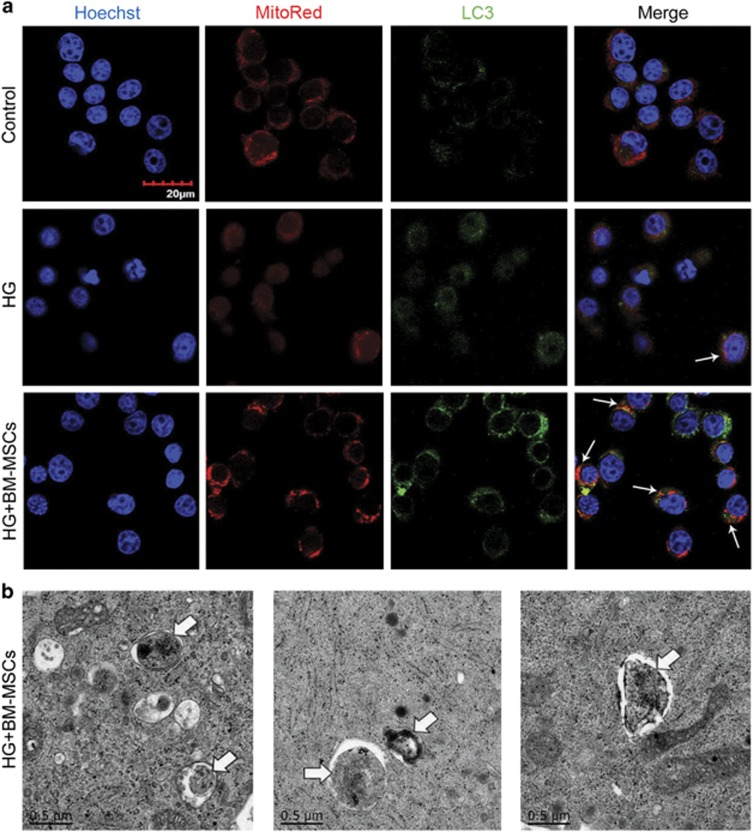
BM-MSCs promoted clearance of dysfunctional mitochondria through enhanced autophagy in chronic HG-treated INS-1 cells. (**a**) Immunofluorescence analysis was used to determine the number of Mito Red-marked mitochondria colocalized with LC3-labeled autophagosomes. White arrows point at the colocalization. Scale bar, 20 *μ*m. (**b**) TEM analysis showed that autophagosomes engulfed damaged mitochondria for degradation in INS-1 cells co-cultured with BM-MSCs. White arrows pointed to mitochondria. Scale bar, 0.5 *μ*m. Magnification: × 40 000. All the data were collected from at least three independent experiments

As removal of dysfunctional mitochondria via autophagy has been reported in many studies,^32, 34^ we opined that BM-MSC-induced recovery of MMP and normalization of ROS levels were perhaps best analyzed in mitochondrial renovation via enhanced autophagy in HG-treated INS-1 cells. Immunofluorescence analysis and TEM analysis were performed to test this hypothesis. Immunofluorescence analysis demonstrated that BM-MSC treatment significantly increased the colocalization of Mito Red-labeled mitochondria and LC3-positive autophagosomes, as compared with little presence of colocalization between mitochondria and autophagosomes in INS-1 cells treated with HG alone, indicating that autophagy enhanced by BM-MSCs may be conducive to remove impaired mitochondria in HG-treated INS-1 cells (Figure 6a). To confirm our immunofluorescence results, TEM analysis was used to evaluate ultrastructural changes in INS-1 cells. The results displayed that autophagosomes or autolysosomes that engulfed impaired mitochondria could be observed in INS-1 cells co-cultured with BM-MSCs. Conversely, the similar ultrastructures were hardly seen in INS-1 treated with HG alone (Figure 6b). Taken together, these findings revealed that BM-MSCs improved mitochondrial function by autophagic removal of impaired mitochondria in chronic HG-treated INS-1 cells.

### BM-MSC infusion ameliorated hyperglycemia and promoted the recovery of pancreatic damage in T2D rats

**Figure 7:**
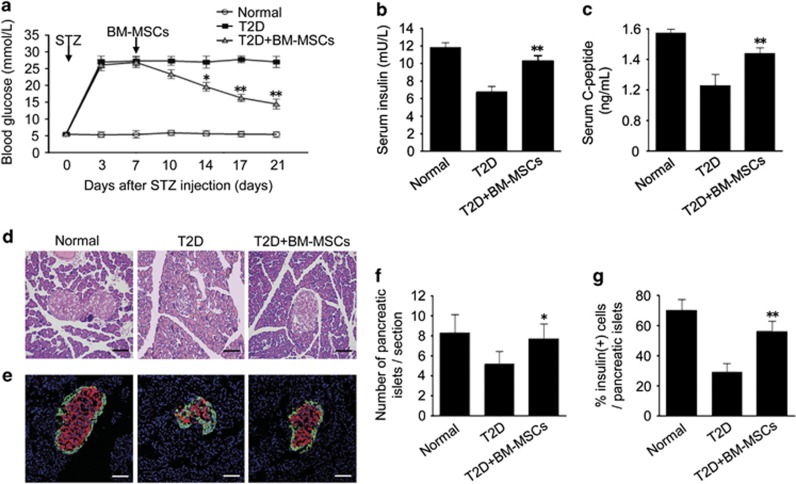
Infusion of BM-MSCs ameliorated hyperglycemia and promoted the restoration of pancreatic islet and *β* cells in T2D rats. Seven days after STZ injection, 2 × 10^6^ BM-MSCs suspended in 0.2 ml physiological saline were injected into T2D rats via tail vein, whereas untreated T2D rats received only 0.2 ml physiological saline. (**a**) Blood glucose level was determined in alert, fasted rats. (**b** and **c**) Insulin and C-peptide levels in fasted and re-fed rats were evaluated by ELISA. (**d**) Pancreas histology was studied in H–E-stained sections. Scale bar, 100 *μ*m. (**e**) Pancreatic islets were characterized by immunofluorescence according to the presence and distribution of insulin- (red) and glucagon-producing (green) cells. Scale bar, 100 *μ*m. (**f**) Pancreatic islets observed in immunofluorescence-stained sections were quantified in T2D rats that received BM-MSC infusion. (**g**) *β* Cells in pancreatic islets were quantified in BM-MSC-treated T2D rats. Values are means±S.E. (*n*=10 rats per group). *P<0.05, **P<0.01 *versus* T2D rats

To evaluate the effects of BM-MSCs on T2D animal models, we performed BM-MSC infusion into the T2D rats induced by a high-fat diet and STZ administration. T2D rats underwent persistent hyperglycemia, accompanied by significant reduction of serum insulin and C-peptide levels. However, BM-MSC-treated type 2 diabetic rats showed a gradual decrease in blood glucose levels, combined with marked increase in serum insulin and C-peptide levels (Figures 7a–c). In addition, histopathologic analysis was performed to examine morphological changes in pancreatic islets. The results showed shrunken pancreatic islets in T2D rats. Nevertheless, BM-MSC infusion restored islet morphology, suggesting the alleviation of morphological destruction in pancreatic islets (Figures 7d and f). We next performed immunofluorescence analysis to further confirm our histopathologic results. Decreased number of insulin-producing cells and reduced pancreatic islets size were observed in T2D rats, but these changes were largely reversed by BM-MSC infusion. Meanwhile, BM-MSC infusion led to a significant increase in the number of pancreatic isles and an elevated ratio of insulin-positive cells per islet as compared with untreated T2D rats (Figures 7e and g). These results together revealed that BM-MSC infusion significantly ameliorated hyperglycemia and promoted restoration of pancreatic islet and *β* cells in T2D rats.

### BM-MSC infusion enhanced autophagic activity in pancreatic *β* cells of T2D rats

**Figure 8:**
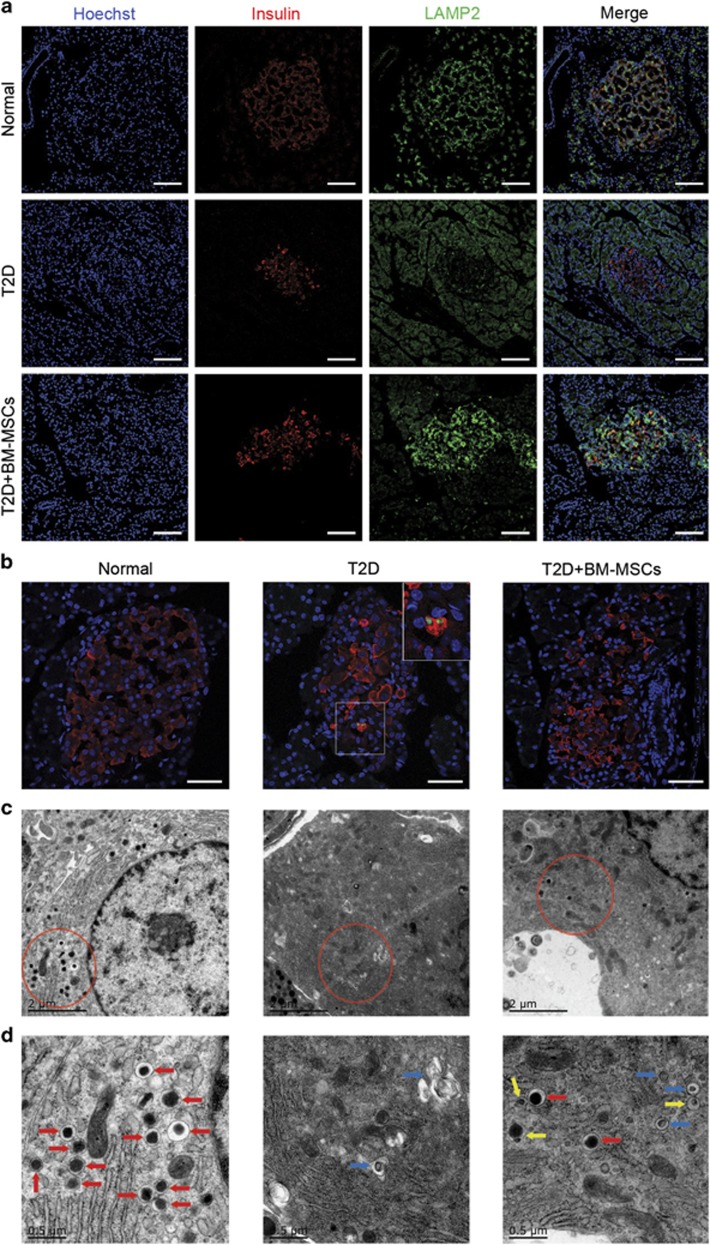
Infusion of BM-MSCs regulated autophagic activity of pancreatic *β* cells in T2D rats. (**a**) LAMP2 expression in *β* cells was assessed by immunofluorescence analysis. Scale bar, 100 *μ*m. (**b**) *β*-Cell apoptosis was detected using TUNEL assay by immunofluorescence analysis according to the presence of insulin- (red) and TUNEL-positive (green) cells. Scale bar, 50 *μ*m. (**c** and **d**) TEM analysis showed ultrastructural changes in pancreatic *β* cells. Blue arrows, autophagosomes; yellow arrows, autolysosomes; red arrows, insulin granules. Scale bar, 2 *μ*m (**c**), 0.5 *μ*m (**d**). Magnification: × 15 000 (**c**), × 40 000 (**d**). *n*=10 rats per group

To extend our findings regarding autophagy to the T2D rat model, we investigated whether the influence of BM-MSCs on restoration of pancreatic *β* cells in T2D rats was in relation to autophagy modulation. Results from immunofluorescence analysis showed marked reduction of LAMP2 expression, combined with increase number of apoptotic *β* cells (TUNEL- and insulin positive) in untreated T2D rats. In contrast, BM-MSC infusion significantly increased LAMP2 expression, and the double-positive cells were rarely seen in BM-MSC-treated T2D rats, indicating that BM-MSC infusion led to enhanced autophagic activity of pancreatic *β* cells with concurrent reduction of *β*-cell apoptosis, which was consistent with our results of *in vitro* study (Figures 8a and b). TEM analysis also displayed that there were few autophagosomes formation, but massive vacuoles accumulation and greatly reduced number of insulin granules in *β* cells of T2D rats. Nevertheless, it was worth noting that BM-MSC infusion significantly enhanced the formation of autophagosomes and autolysosomes, accompanied by increased number of insulin granules in pancreatic *β* cells (Figures 8c and d). We therefore concluded that the significant restoration of pancreatic *β* cells in T2D rats was largely dependent on autophagy augmented by BM-MSC infusion.

## Discussion

Chronic hyperglycemia is recognized as an important initiating factor for continuous deterioration of *β*-cell function and mass in type 2 diabetic patients.^2, 37^ Identifying agents or mechanisms that could protect *β* cells against chronic hyperglycemia-induced injury has become an urgent issue in clinical therapies for T2D mellitus. In this study, our data pointed out that BM-MSCs could alleviate INS-1 cell injury induced by chronic HG and promote restoration of pancreatic *β* cells in T2D rats. Furthermore, we found the potential mechanisms underlying the cytoprotective effects of BM-MSCs were related to autophagy.

Increasing evidence suggested that augment of autophagy was an effective mean to inhibit apoptosis in many disease states. Liu *et al.*^38^ reported that autophagy activation protected renal tubular epithelial cells from urinary protein-induced apoptosis. Cai *et al.*^39^ demonstrated that autophagy stimulated through PKC*α* pathway had a protective role of autophagy in palmitic acid (PA)-induced hepatocytes apoptosis. Similar results were also discovered in pancreatic *β* cells. Han *et al.*^40^ found that autophagy in *β* cells was activated through AMPK pathway as a protective response under chronic HG conditions. Yet at the same time, their results also showed that this activation induced by HG alone seemed insufficient to prevent *β*-cell loss. Our results revealed that there was a slight increase in autophagosomes formation but little presence of fusion between autophagosomes and lysosomes in INS-1 cells treated with HG alone. These results were partially consistent with recent findings concerning suppression of autophagic turnover in *β* cells induced by PA and glucose.^41^ It was not a completed autophagic process if lack of autolysosomes. Defective autophagy decreased *β*-cell mass and function. Mice with *β*-cell-specific autophagy deficiency showed impaired glucose tolerance and reduced insulin secretion.^23, 24^ In addition, recent studies indicated that autophagy deficiency in *β* cells blocked the degradation of human islet amyloid polypeptide (h-IAPP) and increased h-IAPP accumulation, which induced *β*-cell apoptosis and exacerbated diabetes.^42, 43, 44^ In contrast, stimulation of autophagy with the mTORC1 inhibitor rapamycin protected *β* cells against h-IAPP-induced proteotoxicity.^26^ However, rapamycin was proved toxic and deleterious to pancreatic *β* cells and insulin sensitivity of peripheral target tissues when used as an immunosuppressant in transplantation.^45^ Of note, several studies demonstrated that MSCs possessed great capacity to augment autophagy. In Ccl4-injured rat liver model, human placenta-derived MSCs stimulated autophagy in damaged hepatic cells and triggered liver regeneration.^46^ This study also revealed that BM-MSCs promoted the formation of autophagosomes and autolysosomes and thus exert cytoprotective effects on *β* cells, which was consistent with recent reports that treatment with MSCs increased neuronal survival via enhancement of autophagy in both Alzheimer disease models^27^ and parkinsonian models.^28^

Autophagy is a highly conserved self-degradation program that eliminates longevity proteins and damaged organelles in order to maintain cellular homeostasis under various pathological conditions.^17^ Formation and degradation of ubiquitinated protein aggregates was regulated by autophagy during diabetes-induced oxidative stress.^47^ Adequate evidence has demonstrated that chronic HG induces *β*-cell mitochondrial dysfunction combined with considerable intracellular ROS accumulation.^31, 48^ This phenomenon was confirmed in this study as well. Nonetheless, we further discovered that BM-MSCs could improve mitochondrial function and reduce intracellular ROS production through autophagic removal of impaired mitochondria in HG-treated INS-1 cells, which was similar to the results found in rotenone-exposed human neuronal SH-SY5Y cells that rapamycin protected against rotenone-induced injury by facilitating clearance of ubiquitinated proteins and dysfunctional mitochondria.^49^

Our *in vivo* study demonstrated that BM-MSC infusion ameliorated glycemic control and restored pancreatic islets and *β* cells in T2D rats, consistent with our previous studies.^15, 50^ Importantly, we further discovered that the effects of BM-MSCs on *β* cells were associated with autophagy modulation, which has not been published before. BM-MSC infusion upregulated LAMP2 expression and increased the formation of autophagosomes and autolysosomes in pancreatic *β* cells, accompanied by reduced *β*-cell apoptosis. In view of the multiple roles of MSCs *in vivo* and the influence of local microenvironment on MSCs, it seemed difficult and complicated to determine exact mechanisms underlying the protective effects of MSCs on pancreatic *β* cells in T2D rats. Some researchers postulated that MSCs directly participated in damaged *β*-cell repair,^51^ whereas others insisted that the secretive properties of MSCs improved the microenvironment of pancreas and promoted the survival of surrounding cells through secretion of various growth factors and cytokines.^13, 52^ As our *in vitro* experiments were conducted in a Transwell co-culture system that limited the contact just between BM-MSCs and INS-1 cells, and our results also showed BM-MSC-CM could promote autophagy in HG-treated INS-1 cells, we proposed that the significant upregulation of autophagy in *β* cells may be largely caused by secretive properties of BM-MSCs, which could be an indispensable explanation for the protective effects of BM-MSCs on *β* cells in T2D rats. So far TGF-*β* has received growing concern because of its significant impacts on autophagy activation.^53, 54, 55^ TGF-*β* can induce autophagy by upregulating several autophagy-related genes (such as Atg5 and Atg7) through a Smad- and JNK-dependent manner.^56^ As TGF-*β* was also an important growth factor secreted by MSCs, we supposed that TGF-*β* may exert an essential effect on MSCs-induced autophagy, however, which still need to be further explored in future studies.

In summary, this study demonstrated that BM-MSCs enhanced autophagy and thereby protected INS-1 cells against chronic HG-induced injury *in vitro*. Furthermore, infusion of BM-MSCs promoted restoration of pancreatic islets and *β* cells through modulation of autophagy in T2D rats. These results together suggested that BM-MSCs could serve as an ideal candidate of autophagy enhancer and thus exert great cytoprotective effects on *β* cells under chronic HG conditions. This study provides new insights into our understanding of MSC-based therapeutic mechanisms and establishes an important foundation for future clinical use of MSCs in T2D therapy.

## Materials and Methods

### Isolation, culture and identification of BM-MSCs

Fresh BM-derived cells were isolated and harvested from tibia and femurs of 6-week-old male Sprague–Dawley rats as previously described.^50^ The passage 3 cells were identified the characteristics of MSCs by flow cytometer as previously described.^15^

### Preparation of BM-MSC-CM

The CM was collected and condensed from the supernatant of BM-MSCs at passage 3 as previously described.^50^

### Cell culture and processing

BM-MSCs were cultured in DMEM-LG (Hyclone, Logan, UT, USA) medium with 10% fetal bovine serum (FBS; Gibco, Grand Island, NY, USA) and 1% penicillin–streptomycin (Gibco). 208F cells were cultured in DMEM-HG (Hyclone) medium containing 10% FBS and 1% penicillin–streptomycin. BM-MSCs at passage 3 and 208F cells were separately cultured in transwell (Corning, New York, NY, USA) inserts at a density of 5 × 10^3^/cm^2^ for 10–12 h before use.

INS-1 cells were cultured in RPMI 1640 medium (Gibco) supplemented with 10 mmol/l HEPES (Sigma-Aldrich, St. Louis, MO, USA), 1 mmol/l pyruvate (Gibco), 10% FBS, 1% penicillin–streptomycin and 50 *μ*mol/l *β*-mercaptoethanol (Sigma-Aldrich) in a humidified incubator (5% CO_2_ in air at 37 °C). INS-1 cells cultured in the medium containing 11.1 mmol/l glucose were used as the control group, and those cultured in 33.3 mmol/l glucose were used as the HG group. After being exposed to the HG medium for 48 h, INS-1 cells were co-cultured with BM-MSCs or 208F cells for another 12 h before assaying. 3-MA and CQ were both purchased from Sigma-Aldrich and added in the culture medium according to our experimental design.

### Animals and BM-MSC administration

The high-fat diet fed, STZ-induced rat type 2 diabetic model was established as previously described.^50^ This protocol was approved by the medical ethics committee of the Chinese PLA General Hospital.

In all, 2 × 10^6^ BM-MSCs at passage 3 were collected, suspended in 0.2 ml physiological saline and injected into the tail vein of type 2 diabetic rats 7 days after STZ (Sigma-Aldrich) injection. The untreated type 2 diabetic rats were infused with only 0.2 ml physiological saline.

### Cell viability assay and apoptosis assay

INS-1 cells were seeded in 96-well plates at a density of 5 × 10^3^ cells per well. At the end of treatment, INS-1 cells were incubated in fresh medium containing 10 *μ*l of CCK-8 solution (DOJINDO, Kumamoto, Japan) at 37 °C for 3 h. Absorbance was measured at 450 nm using a microplate reader (Thermofisher, Shanghai, China).

Apoptosis incidence in INS-1 cells was quantified using the Annexin V-FITC/PI Apoptosis Detection Kit (BD Biosciences, San Diego, CA, USA) according to the manufacturer's instructions. A total of 10 000 cells were collected each time and the data were acquired by a BD Accuri C6 flow cytometer (BD Biosciences).

### Insulin secretion assay

After all the treatment, INS-1 cells were washed with PBS and then incubated in the Kerbs-Ringer bicarbonate buffer (KRBB, pH=7.4) containing 115 mM NaCl, 4.7 mM KCl, 1.2 mM MgSO_4_, 1.2 mM KH_2_PO_4_, 2.56 mM CaCl_2_, 20 mM NaHCO_3_, 16 mM Hepes and 0.2% BSA at 37 °C for 1 h. Then, the buffer was replaced by fresh KRBB solution supplemented with 5 mM glucose or 25 mM glucose and cells were incubated at 37 °C for another hour. The supernatants were collected and insulin levels were measured by a Ratio Immunity Assay (RIA) kit. Protein content was determined using a BCA Protein Assay Kit (Thermo Scientific, Rockford, IL, USA) and then used to normalize the levels of secreted insulin.

### Western blot analysis

For western blotting, briefly, INS-1 cells were first washed with PBS and lysed with RIPA lysis buffer containing protease inhibitor to extract total protein. After protein quantification, aliquots containing 40 *μ*g of protein were separated by 15% sodium dodecyl sulfate polyacrylamide gel electrophoresis and transferred to PVDF membranes. The membranes were blocked with non-fat milk and incubated with specific primary antibodies overnight at 4 °C. The following primary antibodies and dilutions were used: rabbit anti-cleaved caspase 3 (1 : 500; CST, Boston, MA, USA), rabbit anti-Beclin1 (1 : 1000; CST), rabbit anti-LC3 (1 : 1000; Sigma-Aldrich) and mouse anti-*β*-actin (1 : 2000; Sigma-Aldrich). The next day, the membranes were incubated with secondary antibodies using goat anti-rabbit and goat anti-mouse IgG (HRP). The blots were analyzed with densitometry using Image J software (NIH, Bethesda, MD, USA).

### Green fluorescent protein-LC3

INS-1 cells were transfected with GFP-LC3 plasmid (OriGene Technologies, Inc., Rockville, MD, USA) using Lipofectamine 2000 (Invitrogen, Carlsbad, CA, USA) according to the manufacturer's instructions. GFP-LC3 dots were observed under a fluorescence microscope (Olympus, Tokyo, Japan) with the 488 nm excitation filter. The average number of GFP-LC3 puncta per cell was obtained by counting the puncta among 100 GPF-LC3-positive cells for each sample.

### Immunofluorescence analysis

INS-1 cells and pancreatic sections were rinsed twice in PBS and then incubated with specific primary antibodies overnight at 4 °C. The following antibodies were used: rabbit anti-LC3 (1 : 200; CST), mouse anti-LAMP2 (1 : 200; MBL, Nagoya, Japan), guinea pig anti-insulin (1 : 200; Abcam, San Francisco, CA, USA) and mouse anti-glucagon (1 : 200; CST). The next day, INS-1 cells and pancreatic sections were incubated with secondary antibodies (Invitrogen) using donkey anti-mouse IgG (Alexa Fluor 488; 1 : 500), donkey anti-guinea pig IgG (Alexa Fluor 594; 1 : 500) and goat anti-rabbit IgG (Alexa Fluor 488; Alexa Fluor 594; 1 : 500). Subsequently, the samples were stained with Hoeschst 33342 (Sigma-Aldrich) diluted 1 : 1500 in PBS. Stained cells and tissues were viewed using an Olympus FV1000 confocal laser-scanning microscope with excitation filters at 488 nm (green) and 594 nm (red).

### TEM analysis

INS-1 cell aggregates and pancreatic sections were fixed with 2.5% glutaraldehyde-0.1 M NaH_2_PO_4_/Na_2_HPO_4_ phosphate buffer (pH 7.2) for 2 h at 4 °C. After rinsing three times with phosphate buffer for 30 min, the samples were post-fixed with 1% osmic acid for another 2 h at 4 °C. Then, the samples were dehydrated in ethanol with gradient ascent (50–70–90–100%), replaced by propylene oxide and embedded in Epon 812. Subsequently, semi-thin sections (900 nm) were cut and stained with methylene blue for localization in light microscopy. Ultimately, ultrathin sections (110 nm) were stained with uranyl acetate and lead citrate, and examined under JEM-1400 electron microscope (JEOL, Tokyo, Japan).

### MMP measurements

MMP in INS-1 cells was detected using a dual-emission potential-sensitive fluorescent probe JC-1 (Cayman Chemical, Ann Arbor, MI, USA) according to the manufacturer's instructions. Briefly, INS-1 cells initially were rinsed twice in PBS and incubated with JC-1 for 20 min at 37 °C. Then, the cells were rinsed with PBS again and analyzed with a BD Accuri C6 flow cytometer (BD Biosciences).

### ROS measurements

Intracellular ROS production was detected using an oxygen radical sensitive probe, 2, 7-dichlorodihydrofluorescein diacetate (DCFH-DA) (Molecular Probes, Eugene, OR, USA) according to the manufacturer's instructions. Briefly, INS-1 cells were rinsed with PBS for three times and incubated with DCFH-DA solution for 20 min at 37 °C. Then, INS-1 cells were rinsed with PBS again and detected by a BD Accuri C6 flow cytometer (BD Biosciences).

### Blood glucose, serum insulin and C-peptide measurements

Rats were fasted for 3 h before measuring blood glucose levels. Tail capillary blood glucose levels were monitored throughout the experiments using One Touch Ultra glucose monitor (LifeScan Inc., Milpitas, CA, USA). The levels of serum insulin and C-peptide were measured by enzyme-linked immunosorbent assay (ELISA) (rat insulin, C-peptide ELISA Kit, Millipore, Billerica, MA, USA) according to the manufacturer's instructions.

### Histological analysis and TUNEL staining

After fixation and embedding, paraffin sections (3 *μ*m) of pancreas were stained with hematoxylin–eosin (H–E). Sections were observed by a fluorescence microscope (Olympus).

Frozen sections (7 *μ*m) of pancreas were measured by TUNEL staining using the Roche Cell Death Detection Kit (Roche Diagnostics, Indianapolis, IN, USA). The stained tissues were viewed with a Leica TCS SP8 confocal laser-scanning microscope (Leica, Wetzlar, Germany).

### Statistical analysis

All data are presented as the means±S.E. from at least three independent experiments. Statistical analysis was performed using SPSS version 17.0 (SPSS Inc., Chicago, IL, USA) and comparisons between two groups were measured using Student's *t*-test. Statistical significance was accepted for *P*-values of<0.05.

## Abbreviations

BM-MSC: bone marrow-derived mesenchymal stem cell
HG: high glucose
T2D: type 2 diabetes
BIS: basal insulin secretion
GSIS: glucose-stimulated insulin secretion
LC3: microtubule-associated protein 1 light chain 3
LAMP2: lysosome-associated membrane protein 2
GFP: green fluorescent protein
TEM: transmission electronic microscope
3-MA: 3-methyladenine
CQ: chloroquine
ROS: reactive oxygen species
MMP: mitochondrial membrane potential
