# Supplementary figures and images for: Bone marrow-derived mesenchymal stem cells ameliorate chronic high glucose-induced β-cell injury through modulation of autophagy
Source: Cell Death Dis. 2015 Sep 17;6(9):e1885–. doi: 10.1038/cddis.2015.230 (PMC4650435; doi:10.1038/cddis.2015.230)

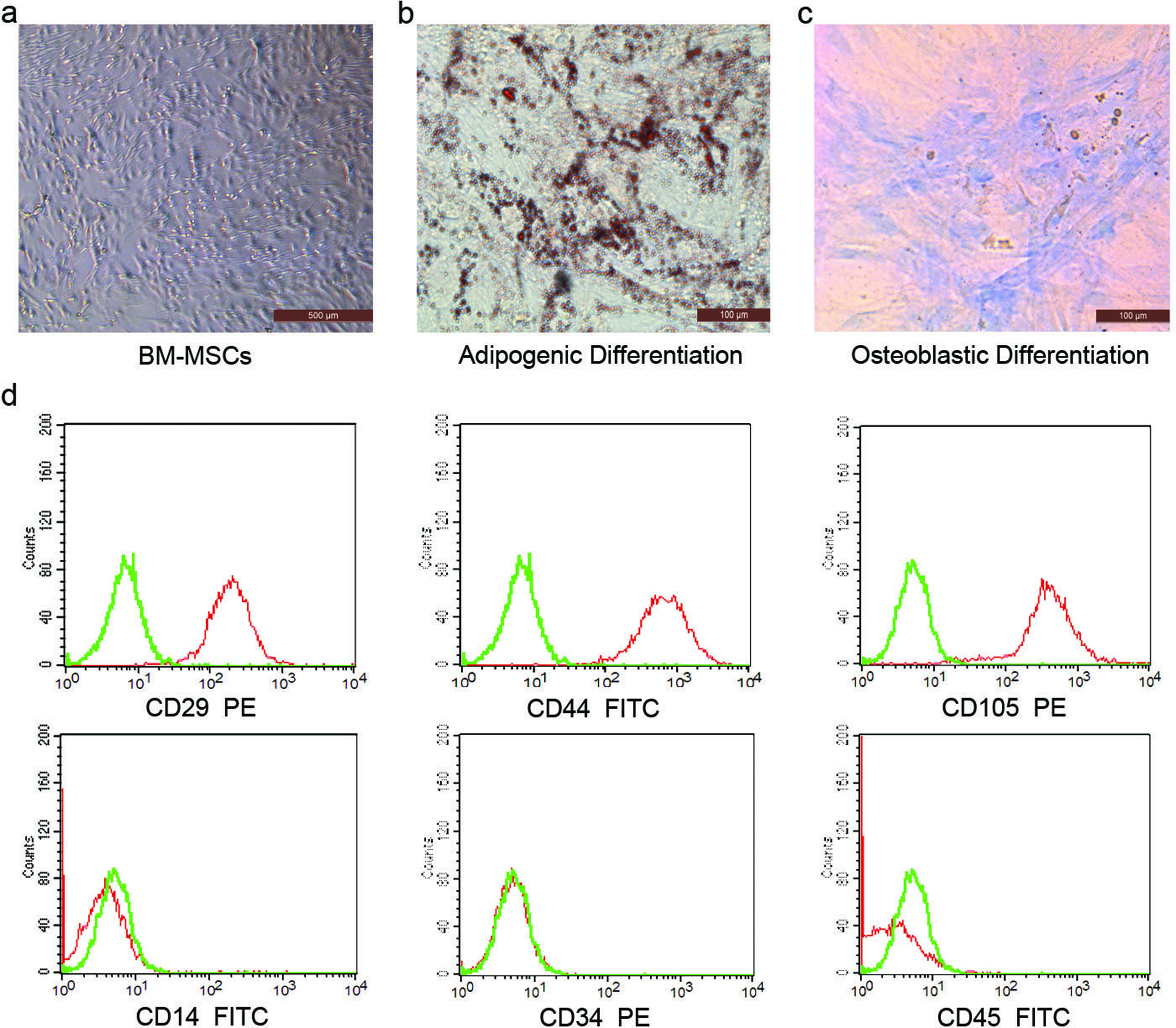

Supplement: Supplementary Figure 1 [file cddis2015230x1.tif]

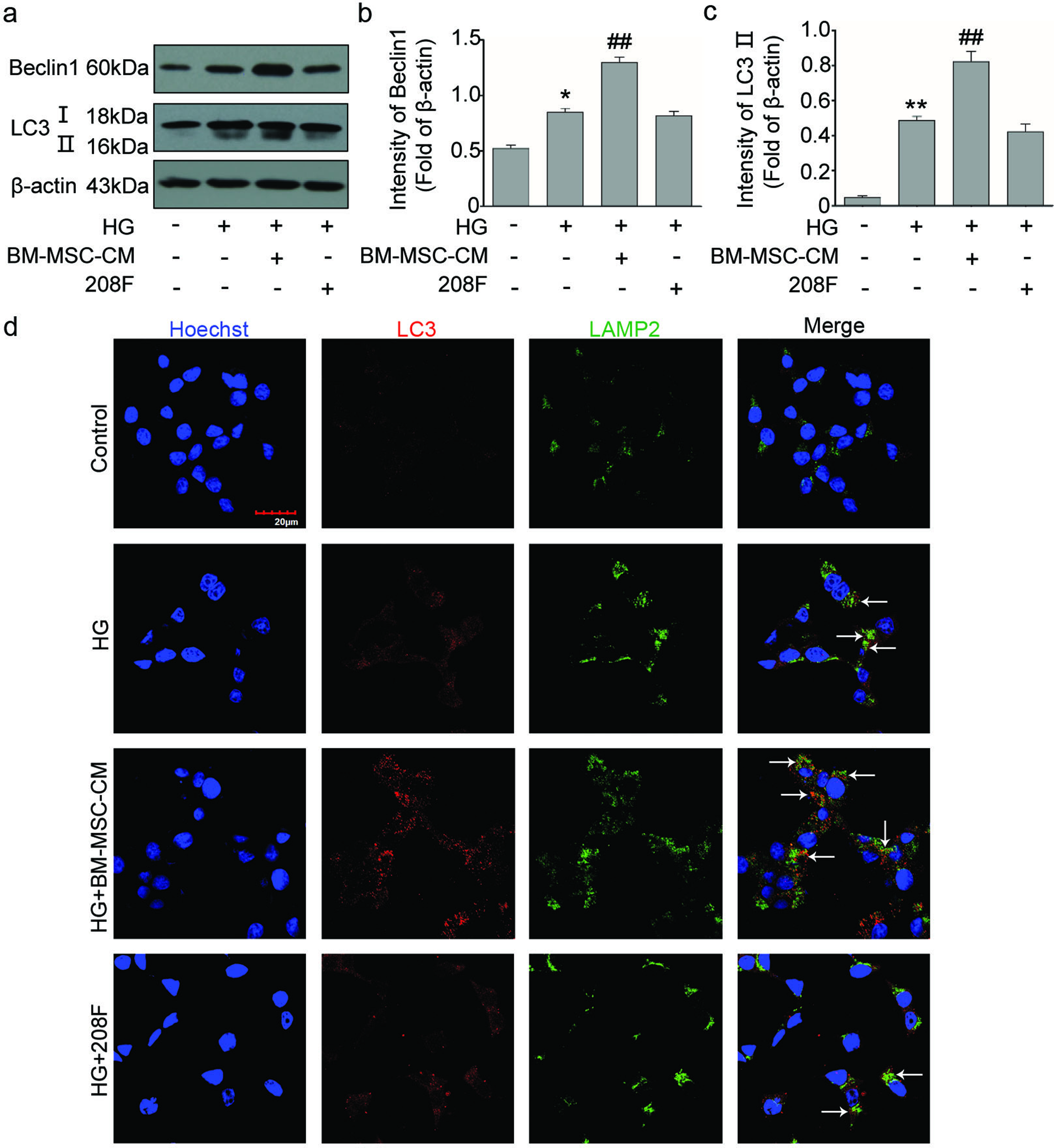

Supplement: Supplementary Figure 2 [file cddis2015230x2.tif]
