# Supplementary material for: Bone marrow-derived mesenchymal stem cells ameliorate chronic high glucose-induced β-cell injury through modulation of autophagy
Source: Cell Death Dis. 2015 Sep 17;6(9):e1885–. doi: 10.1038/cddis.2015.230 (PMC4650435; doi:10.1038/cddis.2015.230)
Supplement: Supplementary Information [file cddis2015230x3.doc]

**Supplementary Figure 1 Identification of BM-MSC characteristics.** (**a**): Morphology analysis under light microscopy showed elongated and spindle-shaped appearance of BM-MSCs at passage three. Scale bar, 500 µm. (**b** and **c**): BM-MSCs differentiated into adipogenic (lipid vesicles) and osteoblastic (alkaline phosphatase; blue) lineages. Scale bar, 100 µm. (**d**): The phenotypes of BM-MSCs were detected by flow cytometer and cells were positive for CD29, CD44, CD105, but negative for CD14, CD34, CD45.

**Supplementary Figure 2 BM-MSC-CM stimulated the autophagic activity in HG-treated INS-1 cells.** (**a-c**): Western blot evaluation of Beclin1 and LC3-II. Protein expression levels were normalized against β-actin. (**d**): Immunofluorescence analysis was used to assess the number of LC3-positive (red) autophagosomes co-localized with LAMP2-labelled (green) lysosomes. White arrows point at the co-localization. Scale bar, 20 µm. *P<0.05, **P<0.01 vs. control group; ^##^P<0.01 vs. HG group.
